# Population structure of *Helicobacter pylori *among ethnic groups in Malaysia: recent acquisition of the bacterium by the Malay population

**DOI:** 10.1186/1471-2180-9-126

**Published:** 2009-06-19

**Authors:** Chin Yen Tay, Hazel Mitchell, Quanjiang Dong, Khean-Lee Goh, Ian W Dawes, Ruiting Lan

**Affiliations:** 1 School of Biotechnology and Biomolecular Sciences, University of New South Wales, Sydney, NSW 2052, Australia; 2 Department of Medicine, Faculty of Medicine, University of Malaysia, Kuala Lumpar, Malaysia

## Abstract

**Background:**

*Helicobacter pylori *is a major gastric bacterial pathogen. This pathogen has been shown to follow the routes of human migration by their geographical origin and currently the global *H. pylori *population has been divided into six ancestral populations, three from Africa, two from Asia and one from Europe. Malaysia is made up of three major ethnic populations, Malay, Chinese and Indian, providing a good population for studying recent *H. pylori *migration and admixture.

**Results:**

Seventy eight *H. pylori *isolates, including 27 Chinese, 35 Indian and 16 Malay isolates from Malaysia were analysed by multilocus sequence typing (MLST) of seven housekeeping genes and compared with the global MLST data. STRUCTURE analysis assigned the isolates to previously identified *H. pylori *ancestral populations, hpEastAsia, hpAsia2 and hpEurope, and revealed a new subpopulation, hspIndia, within hpAsia2. Statistical analysis allowed us to identify population segregation sites that divide the *H. pylori *populations and the subpopulations. The majority of Malay isolates were found to be grouped together with Indian isolates.

**Conclusion:**

The majority of the Malay and Indian *H. pylori *isolates share the same origin while the Malaysian Chinese *H. pylori *is distinctive. The Malay population, known to have a low infection rate of *H. pylori*, was likely to be initially *H. pylori *free and gained the pathogen only recently from cross infection from other populations.

## Background

*Helicobacter pylori *may have infected humans since their origin and currently is believed to infect more than half the population in the world [1,2]. Infection is usually acquired during childhood by intrafamilial transmission and in the majority of cases infection is lifelong unless eradication by antibiotic treatment is undertaken [3,4]. The prevalence of *H. pylori *infection ranges from 25% in developed countries to more than 80% in the developing regions [3,5,6]. *H. pylori *is commonly transmitted from mother to child [3].

*H. pylori *is well known for being highly diverse and recombining frequently. DNA sequence analysis of housekeeping and virulence associated genes all have illustrated the unusually high degree of genetic variability in this species [2,7-12]. Comparison of isolates within a single host sampled over an average of 1.8 years has revealed that an average of ~100 DNA imports occur between bacteria, corresponding to 3% of the genome or 50 kb [11] and by extrapolation from these data, it was predicted that within 41 years half the genome would have been replaced by imports [11]. In comparison, 10–100 million years were needed to replace 60% of the *E. coli *genome [13].

Studies suggest that recombination is rare between isolates from different continents and as such *H. pylori *behaves like a genetic marker of human descent and reflects the human population in which the host spent his/her childhood [2,10,12]. Multilocus sequence typing (MLST) of seven housekeeping genes from several hundred *H. pylori *strains isolated from different geographical, ethnic, and/or linguistic origins showed that *H. pylori *followed human migration out of Africa and identified six *H. pylori *populations which are designated as hpAfrica1, hpAfrica2, hpNEAfrica, hpEurope, hpEastAsia, and hpAsia2 [2,12]. Three of these populations are further divided into subpopulations: hpEastAsia is divided into three subpopulations, hspEAsia, hspAmerind and hspMaori. The hspMaori subpopulation has been isolated exclusively from Maoris and other Polynesians and the hspAmerind from Inuits and Amerinds in North and South America; hpAfrica1 is divided into hspSAfrica and hspWAfrica; hpEurope is divided into Ancestral European 1 (AE1) and Ancestral European 2 (AE2).

Countries with populations of multiple origins provide a good opportunity to further study the population structure of *H. pylori*. Malaysia is composed of three major ethnic populations: Malay (65%), Chinese (26%) and Indian (7.7%) http://www.statistics.gov.my. The majority of Malaysian Chinese migrated from Southern China, the Malaysian Indians from Southern India and the Malays are in general considered natives of Malaysia [14]. The Malaysian Malay population is made up of a mixture of people extant in South East Asia as early as 3000 years ago [15]. However, in modern Malaysia they are now referred to as the Malays [16]. The aboriginal Orang Asli people in Malaysia do not share the same origin as the Malays [17].

*H. pylori *Infection is associated with an increased risk of developing peptic ulcer disease and gastric cancer [18,19] as well as an increased risk of developing primary non-Hodgkin's lymphomas of the stomach (MALT lymphoma) [20]. Previous studies have shown that the Indian ethnic group has the highest rate of *H. pylori *infection (68.9–75%), followed by the Chinese (45–60%) and the Malay the lowest (8–43%) [21,22]. This difference of prevalence was also found in children [23]. Interestingly the three populations have different rates of gastric cancer. While the Malaysian Chinese population has a high incidence the Malaysian Indian population has a low incidence [24]. The phenomenon of high prevalence of *H. pylori *but low incidence of gastric cancer has been dubbed the "Indian Enigma" [24]. A better understanding of the population structure of *H. pylori *in these ethnic populations is clearly needed to order to elucidate the differences in infection rates and disease severity. We used MLST to analyse *H. pylori *isolates obtained from the three ethnic groups in Malaysia. We show the similarity between the Malay and the Indian *H. pylori *isolates and the diversity between the Malaysian Indian *H. pylori *population identified in this study and the Indian Ladakh *H. pylori *population identified by Linz *et al*. [2].

## Results

### Nucleotide diversity of the housekeeping genes

Fragments of seven housekeeping genes,*atpA *(566 bp), *efp *(350 bp), *mutY *(361 bp), *ppa *(338 bp), *trpC *(396 bp), *ureI *(525 bp), and *yphC *(450 bp), with a total length of 2,982 bp were sequenced from 78 Malaysian *H. pylori *isolates, including 27 Chinese, 16 Malay and 35 Indian isolates. MLST data of 423 isolates comprising of isolates from two studies by Achtman's group [2,12] available at the time of analysis were extracted from the *H. pylori *MLST database http://pubmlst.org/helicobacter/ and included in the analysis with data from this study. The level of nucleotide diversity between populations and between genes is shown in Table 1. The most diverse gene was *trpC *in all except the Malaysian Chinese population with the highest diversity at nearly 7.6% while the least diverse gene was *atpA *at 2.6%. The three ethnic populations showed different levels of diversity with the Chinese population the lowest while the Indian and Malay populations were similar. All ethnic groups had lower level of variation than the global population as a whole.

**Table 1:** Sequence variation

Gene	Size (bp)	Diversity (%)				Population segregation sites			
		Chinese (27)	Indian (35)	Malay (16)	Global (492)	hspEAsia vs hspMaori	hspEAsia vs hspAmerind	hspIndia vs hspEAsia	hspIndia vs hspLadakh

*atpA*	566	1.77	1.61	2.22	2.62	5	4	5	4
*efp*	350	1.95	2.38	3.13	3.34	4	1	6	3
*mutY*	361	3.62	4.85	4.49	6.5	8	7	9	7
*ppa*	338	1.76	2.24	2.16	3.22	1	1	1	0
*trpC*	396	3.35	6.78	6.91	7.6	9	16	16	16
*ureI*	525	2.08	2.39	2.66	3.21	9	9	8	5
*yphC*	450	2.34	3.79	3.87	4.84	10	4	8	6

All seven	2,980	2.37	3.35	3.55	4.33	39	32	48	27

### STRUCTURE analysis

**Figure 1:**
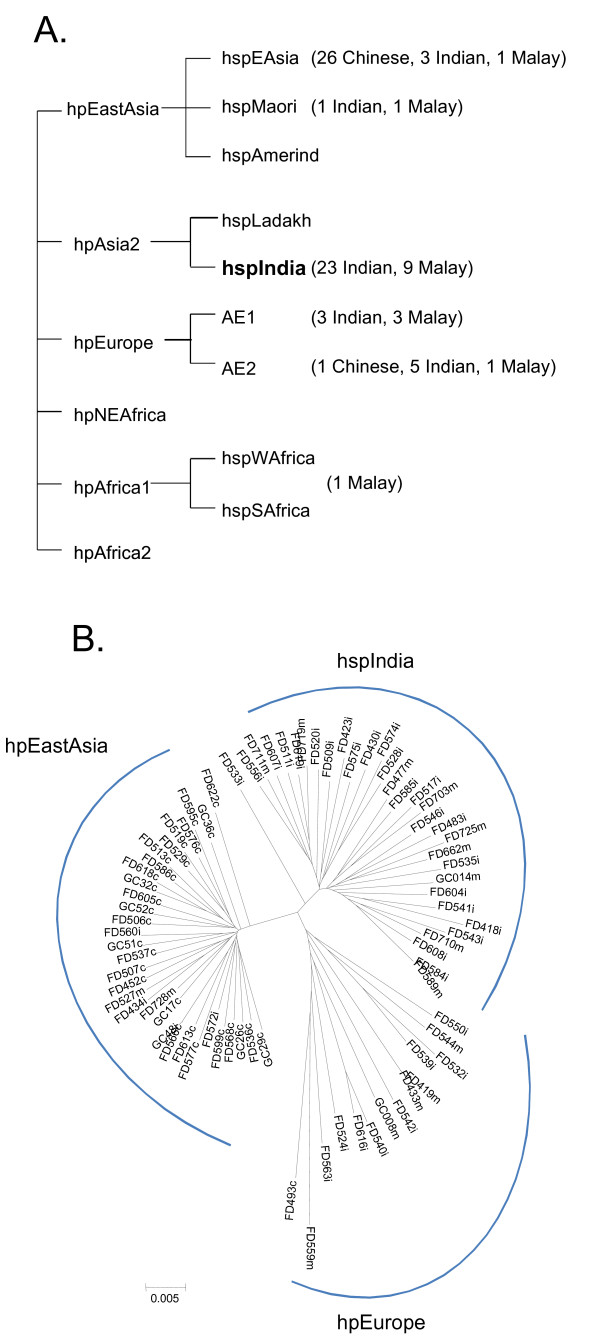
**Population and phylogenetic structure of the Malaysian isolates**. A) Ancestral populations and population assignment of the Malaysian isolates. The division into populations and subpopulations according to Falush *et al*. [12] and Linz *et al*. [2] with the new subpopulation identified in this study in bold. The number of isolates from this study falling into each subpopulation or population is shown in brackets. B) Neighbour joining tree of the Malaysian isolates.

To determine the relationship of the Malaysian *H. pylori *isolates and the global isolates, we analysed our MLST data together with the global data using the Bayesian statistics tool, STRUCTURE [25], which was previously used to divide global *H. pylori *isolates into six ancestral populations, designated as hpAfrica1, hpAfrica2, hpNEAfrica, hpEurope, hpEastAsia and hpAsia2 [2,12]. The Malaysian *H. pylori *isolates were found to fall into four of the six known populations (Fig. 1A). Twenty three Indian and nine Malay isolates were grouped with hpAsia2; 26 Chinese, four Indian and two Malay isolates grouped with hpEastAsia; one Chinese, eight Indian and four Malay isolates grouped with hpEurope; and one Malay isolate grouped with hpAfrica1 (Fig. 1A). Phylogenetic analysis using the Neighbour joining algorithm as shown in Figure 1B divided the isolates into three clusters, consistent with the STRUCTURE analysis.

**Figure 2:**
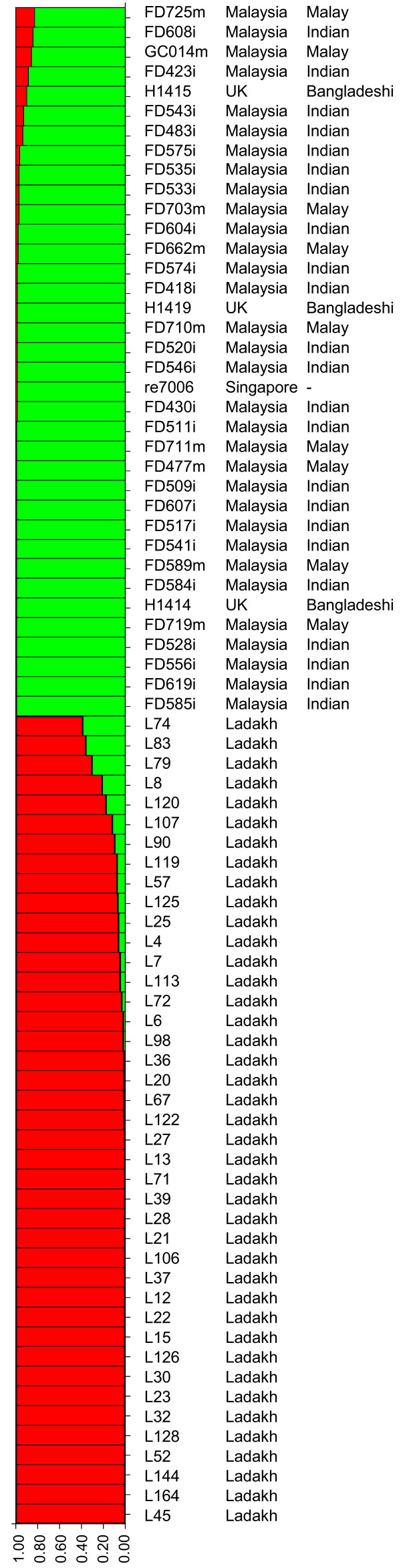
**Division of hpAsia2 into subpopulations by STRUCTURE analysis**. The two subpopulations, hspLadakh (red) and hspIndia (green) and assignment of isolates were shown. Each horizontal bar represents an isolate with isolate names and population and/or ethnic origin shown on the right. All Malaysian isolates were from this study while other isolates from the global MLST data. Mosaic colours for an isolate indicate mixed population origin from respective populations of matching colour. Y-axis represents percentage of population assignment.

Since some populations can be further divided into subpopulations (Fig. 1A) [2,12], we used the same approach to further classify the Malaysian isolates into subpopulations. For the Malaysian isolates in the hpEastAsia population, the majority (26 Chinese, three Indian and one Malay) fell into hspEAsia except for two isolates (one Indian and one Malay) falling into the hspMaori subpopulation. hpAsia2 had previously no subpopulations. There were 77 isolates in hpAsia2 including 32 isolates from this study and 41 Ladakh isolates. Our STRUCTURE analysis divided these 77 isolates into two subpopulations (Fig. 2). All 41 Ladakh isolates were grouped as one subpopulation while the remaining 36 isolates including 32 Malaysian Indian and Malay isolates from this study, one Singapore isolate and three UK isolates (Bangladesh origin) grouped together as another (Fig. 2). Therefore we named the two subpopulations as hspLadakh and hspIndia respectively. For the 13 Malaysian isolates falling into hpEurope, three Indian and three Malay isolates belonged to AE1 while one Chinese, five Indian and one Malay isolate belonged to AE2.

### Identification of polymorphisms distinguishing the subpopulations

**Figure 3:**
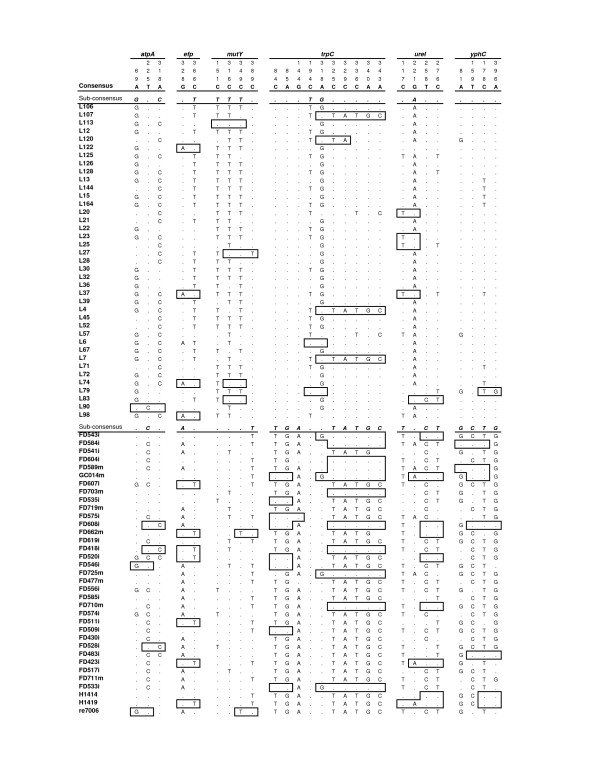
**Population segregation sites between hspIndia and hspLadakh**. The overall consensus is shown at the top. Subpopulation consensus is shown above each subpopulation. Boxed sites shown are segments with at least two identical population segregation sites to the other population. Ladakh isolates (with names starting L) on the top panel and isolates from this study at the bottom panel. Genes and site positions were shown at top (reading vertically). *ppa *is not shown as it has no population segregation sites.

Based on above STRUCTURE analysis, we reasoned that there must be informative bases that support the division of the subpopulations. To identify these bases, we performed site-by-site pairwise comparisons between subpopulations using Fisher's exact test at a significance level of 0.05 with Dunn-Sidak correction for multiple site comparisons. We examined five subpopulations in four comparisons, hspLadakh versus hspIndia, hspEAsia versus hspIndia, hspEAsia versus hspMaori, and hspEAsia versus hspAmerind subpopulations. Out of the 413, 377, 362 and 377 informative sites in the four pairwise comparisons, 27, 48, 39 and 32 sites respectively support the population divisions and we define these sites as population segregation sites (PSSs) (Table 1 and Fig. 3). The gene containing the most PSSs was *trpC *which was also the most variable gene while the gene carrying the fewest number of PSSs was *ppa *with zero or one site. The sites supporting one subpopulation division may not support another population division.

The patterns of the PSSs also provided further insight into recombination between populations. STRUCTURE analysis showed that in all subpopulations there were isolates with genes from other populations but the analysis did not identify which gene contributes to the mosaic genetic background. As shown in Figure 3 for hspIndia and hspLadakh comparison, the PSSs clearly showed the origin of some imported genes. Some involved the whole gene while others only involved segments of a gene. Many of these recombinational events must have occurred in the original population in India. The identification of the PSSs supports the results of STRUCTURE analysis which showed 8.9 to 33.2% imports and for the first time allowed us to identify the ancient alleles or sites in the populations concerned. The total number of PSSs between populations also reflects the distance between them. The more distantly related populations carry more segregating sites.

### Isolates with identical alleles

*H. pylori *has been reported to be clonal only over a short period of time [11] and thus identical alleles among isolates is expected to be rare when sampling a large population. Interestingly, among the 78 Malaysian isolates analysed, 14 isolates had one or more identical alleles to other isolates. Two pairs of isolates, FD584i/FD589i, and FD419m/FD433m were identical in all seven genes; one pair of isolates, GC48i and FD566c, shared six identical genes; two pairs of isolates, FD539i and FD523i, and FD616i and FD540i share four identical genes; another two pairs of isolates, FD529c and FD519c, and FD556i and FD574i shared two identical genes and seven sets of isolates of 2–5 isolates shared one identical gene. Most of the identical genes were shared among the same ethnic population. However, we did observe that some genes were shared by different ethnic populations, most of which share only one identical gene. An Indian isolate (GC48i) shared six identical genes with a Chinese isolate (FD566c) and another Indian isolate (FD560i) had an identical gene with three Chinese isolates (FD586c, GC26c and GC52c).

We extended our analysis to include the 423 global isolate data to screen for identical genes that were shared globally. Fourteen pairs of isolates had all seven genes identical. There were 12, 6, 14, 15, 20, 35 sets with at least two isolates in each set sharing exclusively 6, 5, 4, 3, 2, and 1 identical alleles respectively. In a small number of cases a single isolate shared a subset of alleles with isolates that had a higher number of identical alleles these isolates were excluded. Isolates shared the most alleles in the *efp *gene and the least in *ureI *and *yphC*.

## Discussion

### Population Structure of *H. pylori *among Malaysian Populations

*H. pylori *has been shown to have migrated with its host out of Africa [2,12] and population differentiation is seen clearly in different regions of the world. However no studies have looked at recent *H. pylori *migration histories. Malaysia has a history of human immigration divided into three major waves, the earliest human settlement by the Orang Asli people – the Malay aborigines, the migration of current Malays 3000 years ago, and the mid-nineteenth century migration of Chinese and Indians. There is no data on *H. pylori *infection in the Orang Asli people, but good studies of the other three major ethnic populations are available [22,23,26]. The *H. pylori *infection rate and disease severity are different among the three ethnic populations. This population mixture in Malaysia provided a good opportunity to determine the *H. pylori *population admixture and to enhance our understanding of differences in infection rate and disease severity. We have shown in this study that the isolates recovered from the Malaysian *H. pylori *population belong to three of the known *H. pylori *ancestral populations, hpEastAsia, hpAsia2 and hpEurope. The *H. pylori *isolates from the Chinese and Indian individuals were divided along their ethnic origins. Surprisingly the Malay isolates did not have a separate origin which is discussed below. There were six Indian isolates having Chinese *H. pylori *ancestry but none the reverse.

The population divisions identified in the current study are supported by the distribution of the *cagA *phosphorylation motif EPIYA [27] and *vacA *alleles [26] reported in these populations. The predominant EPIYA motif in the Malaysian Chinese population has been shown to be ABD (87.8%) while the predominant type in both the Malaysian Indian and the Malay populations is ABC with a frequency of 60.5% and 46.2% respectively. For *vacA*, the predominant genotype has been reported to be s1a among the Malaysian Malay (76.6%) and Indian populations (71.0%), and s1c among the Malaysian Chinese population (66.1%) [26]. Data from these two genes confirm our observation that the Malay *H. pylori *population is more similar to Indian than to Chinese population.

It has been suggested that the combined effect of high levels of recombination and diversity does not allow phylogenetic analysis of *H. pylori *isolates [2,12] and also implies that one would not expect to find any identical alleles to be recovered from the population unless they are from related hosts. However for the first time, we uncovered isolates with identical alleles, ranging from one to seven alleles, within and between the three Malaysian populations. The available patient medical information showed that these isolates were not from related hosts. We also found isolates with up to seven identical alleles present in the global MLST data, which was not described previously. The recovery of isolates with identical alleles indicates that the frequency of recombination may be lower and hence clones may be more stable than previously thought. We suggest that isolates with even one identical allele are likely to be related by decent rather than recent recombination involving that gene. Thus it may be possible to determine relationships of isolates if more genes are sequenced.

### The origin of Malay *H. pylori*

The Malay *H. pylori *population did not form a group of its own. The majority (nine of the 16 isolates studied) belong to the same group as the Indian isolates. Clearly the Malay isolates share the same origin as the Indian isolates. This conclusion has a number of implications for the origin of the Malay people and Malay *H. pylori*. Previous studies have shown that *H. pylori *follows the human route of migration and reflects human ancestry. However there is no evidence that ancestral Malays migrated from India. Currently there are two theories for the origins of Malay [28], one being of Southeast Asian origin, specifically sharing common ancestry with the Thais, the Laotians and the Cambodians while the other of Southern China origin through migration to Taiwan, then outwards to the Philippines, Borneo, Indonesia and Malaysia. The latter theory is supported by language origins while the former is supported by genetic evidence [28]. Neither supports Malays sharing direct common ancestry with Indians. Therefore for the Malay population, the ancestry of *H. pylori *does not reflect human ancestry as in other populations.

This raises the question as to what happened with the original Malay *H. pylori *since the human population undoubtedly carried the bacterium before migrating out of Africa. Studies showed that the *H. pylori *infection rate in the Malay population is much lower than that in the Indian population [22]. It is therefore likely that the Malay population was initially free of *H. pylori *and that the *H. pylori *in the current Malay population has only recently been acquired from the Malaysian Indian community. It is possible that the Malay population lost its original *H. pylori *[29]. However loss of *H. pylori *in modern populations is associated with improved living standards and this would be unlikely to be a plausible explanation for the initial loss of *H. pylori *in the Malay population.

While the Indian and Chinese populations have a small percentage of isolates from populations other than their ancestral populations (ie hspIndia and hspEAsia respectively), the Malay population has a much higher proportion of isolates (7 of the 16 isolates studied, 43.75%) from populations other than hspIndia (see discussion below). This adds support to the hypothesis that the Malay population was initially free from *H. pylori *and that these isolates were directly imported from other populations recently. The higher proportion of Malay isolates from the Indian population than from the Chinese population suggests that there has been greater direct interaction between the Malay and Indian populations than between the Malay and Chinese populations. Reduced interaction between the Malays and Chinese may relate to factors such as incompatibility in food sources (eg pork) [30] which may have partly constrained the transmission of *H. pylori *from the Chinese to the Malay population.

Another potential source of *H. pylori *for non-aboriginal Malays is the Orang Asli population, who originated from early human migration out of Africa. The Orang Asli is likely to have taken the "Southern Route" into South East Asia to reach Malaysia by traveling along the Indian Ocean Coast line 50–65,000 years ago [31-33]. Therefore the Orang Asli *H. pylori*, if it exists, may share common ancestry with the Indian *H. pylori*, leading to the observed similarity of Malay isolates to Indian isolates. However given that other earlier *H. pylori *populations such as the Maori and American Indian populations can be readily identified [12], one would expect that the Orang Asli *H. pylori *population would be unique and identifiable after such a long period of separation, arguing against acquisition from Orang Asli population and in favour of acquisition from the Indian population.

### Flow of *H. pylori *genes/genotypes among the Malaysian population and from other populations

Apart from the Malay population who appear to have gained the majority of its *H. pylori *isolates from the Indian population as discussed above, there was also gene flow from other populations. In particular the Indian and Malay populations have higher levels of inflow of genes. Thirteen of the 51 (25.5%) Malaysian Indian/Malay isolates were found grouped with the hpEurope population: six isolates grouped with AE1 and seven with AE2 (Additional file 1). One Malay isolate was found to be grouped with hpAfrica1, and one Indian and one Malay isolates grouped with hspMaori. The Malaysian Chinese population seems to have little inflow of genes from other populations with the exception of one Chinese isolate which grouped with AE2. The low frequency of Chinese isolates with other population affinity indicates that this isolate was more likely to have been acquired by its current or most recent host directly from an AE2 *H. pylori *host.

In contrast, the Indian/Malay isolates with ancestral European history (Table 2) are more likely to represent greater heterogeneity in the Indian/Malay *H. pylori *population and not direct transmission of isolates from the current European population or from early British or Portuguese colonization as these strains have genes from the Indian *H. pylori *gene pool. These isolates contain 8% to 40% hspIndia genes based on STRUCTURE analysis. By population segregation sites, 14 segments with at least two PSSs identical to the Indian/Malay population were identified (data not shown). Three isolates have one identical (PSSs) allele (FD542i in *atpA*, FD550i in *mutY*, FD540i in *ureI*). In contrast, the only Chinese isolate (FD493c) with a European ancestry showed almost no signal of Indian or Chinese ancestry. Such a diversity of isolates in the Malaysian population is interesting and warrants further studies.

**Table 2:** List of strains and population affinity based on STRUCTURE analysis

Strain ID	Disease	Ethnic origin	Population affinity (%)^$^	Population	subpopulation
FD418i	Functional dyspepsia	Indian	81	hpAsia2	hspIndia
FD423i	Functional dyspepsia	Indian	72	hpAsia2	hspIndia
FD430i	Functional dyspepsia	Indian	78	hpAsia2	hspIndia
FD434i	Functional dyspepsia	Indian	96	hpEastAsia	hspMaori
FD483i	Functional dyspepsia	Indian	84	hpAsia2	hspIndia
FD509i	Functional dyspepsia	Indian	77	hpAsia2	hspIndia
FD511i	Functional dyspepsia	Indian	75	hpAsia2	hspIndia
FD517i	Functional dyspepsia	Indian	78	hpAsia2	hspIndia
FD520i	Functional dyspepsia	Indian	83	hpAsia2	hspIndia
FD524i	Functional dyspepsia	Indian	54	hpEurope	AE2
FD528i	Functional dyspepsia	Indian	77	hpAsia2	hspIndia
FD532i	Functional dyspepsia	Indian	46	hpEurope	AE1
FD533i	Functional dyspepsia	Indian	71	hpAsia2	hspIndia
FD535i	Functional dyspepsia	Indian	88	hpAsia2	hspIndia
FD539i	Functional dyspepsia	Indian	62	hpEurope	AE1
FD540i	Functional dyspepsia	Indian	67	hpEurope	AE2
FD541i	Functional dyspepsia	Indian	81	hpAsia2	hspIndia
FD542i	Functional dyspepsia	Indian	40	hpEurope	AE1
FD543i	Functional dyspepsia	Indian	86	hpAsia2	hspIndia
FD546i	Functional dyspepsia	Indian	81	hpAsia2	hspIndia
FD550i	Functional dyspepsia	Indian	48	hpEurope	AE2
FD556i	Functional dyspepsia	Indian	82	hpAsia2	hspIndia
FD560i	Functional dyspepsia	Indian	90	hpEastAsia	hspEAsia
FD563i	Functional dyspepsia	Indian	54	hpEurope	AE2
FD572i	Functional dyspepsia	Indian	97	hpEastAsia	hspEAsia
FD574i	Functional dyspepsia	Indian	82	hpAsia2	hspIndia
FD575i	Functional dyspepsia	Indian	86	hpAsia2	hspIndia
FD584i	Functional dyspepsia	Indian	85	hpAsia2	hspIndia
FD585i	Functional dyspepsia	Indian	71	hpAsia2	hspIndia
FD604i	Functional dyspepsia	Indian	88	hpAsia2	hspIndia
FD607i	Functional dyspepsia	Indian	84	hpAsia2	hspIndia
FD608i	Functional dyspepsia	Indian	83	hpAsia2	hspIndia
FD616i	Functional dyspepsia	Indian	50	hpEurope	AE2
FD619i	Functional dyspepsia	Indian	79	hpAsia2	hspIndia
GC48i	Functional dyspepsia	Indian	95	hpEastAsia	hspEAsia
FD452c	Functional dyspepsia	Chinese	91	hpEastAsia	hspEAsia
FD493c	Functional dyspepsia	Chinese	63	hpEurope	AE2
FD506c	Functional dyspepsia	Chinese	97	hpEastAsia	hspEAsia
FD507c	Functional dyspepsia	Chinese	96	hpEastAsia	hspEAsia
FD513c	Functional dyspepsia	Chinese	86	hpEastAsia	hspEAsia
FD519c	Functional dyspepsia	Chinese	89	hpEastAsia	hspEAsia
FD529c	Functional dyspepsia	Chinese	97	hpEastAsia	hspEAsia
FD536c	Functional dyspepsia	Chinese	95	hpEastAsia	hspEAsia
FD537c	Functional dyspepsia	Chinese	92	hpEastAsia	hspEAsia
FD566c	Functional dyspepsia	Chinese	95	hpEastAsia	hspEAsia
FD568c	Functional dyspepsia	Chinese	93	hpEastAsia	hspEAsia
FD576c	Functional dyspepsia	Chinese	98	hpEastAsia	hspEAsia
FD577c	Functional dyspepsia	Chinese	85	hpEastAsia	hspEAsia
FD586c	Functional dyspepsia	Chinese	93	hpEastAsia	hspEAsia
FD595c	Functional dyspepsia	Chinese	83	hpEastAsia	hspEAsia
FD599c	Functional dyspepsia	Chinese	94	hpEastAsia	hspEAsia
FD605c	Functional dyspepsia	Chinese	93	hpEastAsia	hspEAsia
FD613c	Functional dyspepsia	Chinese	95	hpEastAsia	hspEAsia
FD618c	Functional dyspepsia	Chinese	90	hpEastAsia	hspEAsia
FD622c	Functional dyspepsia	Chinese	83	hpEastAsia	hspEAsia
GC17c	Gastric Cancer	Chinese	89	hpEastAsia	hspEAsia
GC26c	Gastric Cancer	Chinese	92	hpEastAsia	hspEAsia
GC29c	Functional dyspepsia	Chinese	95	hpEastAsia	hspEAsia
GC32c	Gastric Cancer	Chinese	89	hpEastAsia	hspEAsia
GC36c	Gastric Cancer	Chinese	88	hpEastAsia	hspEAsia
GC51c	Gastric Cancer	Chinese	94	hpEastAsia	hspEAsia
GC52c	Gastric Cancer	Chinese	95	hpEastAsia	hspEAsia
FD419m	Functional dyspepsia	Malay	56	hpEurope	AE1
FD433m	Functional dyspepsia	Malay	57	hpEurope	AE1
FD477m	Functional dyspepsia	Malay	73	hpAsia2	hspIndia
FD527m	Functional dyspepsia	Malay	88	hpEastAsia	hspMaori
FD544m	Functional dyspepsia	Malay	59	hpEurope	AE1
FD559m	Functional dyspepsia	Malay	69	hpAfrica1	unknown
FD589m	Functional dyspepsia	Malay	86	hpAsia2	hspIndia
FD662m	Functional dyspepsia	Malay	86	hpAsia2	hspIndia
FD703m	Functional dyspepsia	Malay	83	hpAsia2	hspIndia
FD710m	Functional dyspepsia	Malay	83	hpAsia2	hspIndia
FD711m	Functional dyspepsia	Malay	73	hpAsia2	hspIndia
FD719m	Functional dyspepsia	Malay	75	hpAsia2	hspIndia
FD725m	Functional dyspepsia	Malay	82	hpAsia2	hspIndia
FD728m	Functional dyspepsia	Malay	97	hpEastAsia	hspEAsia
GC008m	Functional dyspepsia	Malay	53	hpEurope	AE2
GC014m	Gastric Cancer	Malay	89	hpAsia2	hspIndia

$ Denotes % genes allocated to the given population by STRUCTURE analysis

There are two other possible sources of genetic heterogeneity in the Indian *H. pylori*: century-old importation and earlier common ancestry. While the colonisation of India from as early as the fifteenth century by the Portuguese and later by the British Empire [34] may have contributed directly to the Indian *H. pylori *gene pool from the European population. This explanation does not reconcile well with the observation by Wirth *et al*. [35] that in the Ladakh population genes of European ancestry were found despite the population being in a more pristine region. It has previously been suggested that AE1 originated in Central Asia because it shares phylogenetic signals with isolates from Estonia, Finland and Ladakh [12,36]. Since the ancestors of Malaysian Indians and the Ladakhis resided in the same region and their *H. pylori *belong to hpAsia2, it seems likely that this biased share of the gene pool by the Malaysian Indian/Malay isolates with the AE1 subpopulation is due to an earlier common ancestry. Our findings are consistent with the studies of Wirth *et al*. [35], Linz *et al*. [2] and Devi *et al*. [19] that *H. pylori *in the Indian population is more heterogeneous in origin, reflecting perhaps both earlier common ancestry and recent imports.

### Division of hpAsia2 into subpopulations

The hpAsia2 population was initially defined based on isolates from Ladakh in Northern India, which represents the west/middle Asia population. Since the Malaysian Indian population is known to have originated from India, the Malaysian Indian isolates were initially not expected to be distinguishable from the Ladakh population. However, we have shown that the two populations can be divided within hpAsia2 as subpopulations, hspLadakh and hspIndia (Fig. 2). A total of 27 (or 0.91%) segregating sites among the seven housekeeping genes were identified to separate the two subpopulations. There is however considerable gene flow between the two populations. Identical alleles as defined by the PSSs can be treated as recombination that occurred in the more distant past. These alleles are present in three genes (*atpA*, *efp *and *ureI*). Further many segments with at least two identical PSSs are present in three other genes (*mutY, trpC *and *yphC*; Fig. 3). Note that *ppa *has no PSSs. These results suggest that there is considerable population admixture in the earlier history of the Indian population. A recent study of the Indian population sequenced 23 isolates by MLST but the sequences are shorter [19]. STRUCTURE analysis of combined data from our Malaysian Indian isolates, Ladakh isolates and these 23 Indian isolates using k = 2 populations and found that the Malaysian Indian isolates grouped together with the Indian isolates while the Ladakh isolates were separate. However, when k = 3 populations were used, the two sets of Indian isolates were separated (data not shown). This suggests that the two Indian populations overlap but are distinctive. The Malaysian Indian *H. pylori *population may have differentiated further from the Indian *H. pylori *population from India, although it is also possible that the difference between the two *H. pylori *populations reflects regional differences in India as the Malaysian Indians mainly came from South India.

## Conclusion

This study has shown that the Malaysian *H. pylori *isolates can be differentiated into three populations using MLST, being hpEastAsia, hpAsia2 and hpEurope. Interestingly the Malay population was shown to carry *H. pylori *isolates of Indian origin. The infection rate of *H. pylori *among the Malay population is low in comparison to the Malaysian Indian population [22]. In western countries a low or reduced rate of *H. pylori *infection is attributed to high or improved hygiene standard [3]. However this factor does not account for differences between the Malay and the other two populations [21,22]. Therefore the Malay population was likely to be initially *H. pylori*-free and has acquired *H. pylori *only recently from the Indian population. Thus the low *H. pylori *infection rate in the Malay population may be due to low cross infection rate from another population.

The Malaysian Indian/Malay isolates were found to differ from the Ladakh isolates from India and in fact formed a new subpopulation, hspIndia. Clearly there are more subpopulations of *H. pylori *and populations can be divided at a finer scale when more isolates are used or more geographical regions are sampled. More extensive worldwide surveys will help us further understand the evolution and population structure of *H. pylori*, an organism that has impacted more than half of the world's population and continues to pose great risk to human health because of its association with gastric cancer and MALT lymphoma. Genetic heterogeneity of the bacterium within a host population as shown in this study should be taken into account when studying the epidemiology and pathogenesis of *H. pylori *since there is clearly variation in incidence and severity of the disease in different populations.

## Methods

### Source of gastric biopsies and culture of *H. pylori *isolates

Gastric biopsies were collected as part of a large-scale gastric cancer study conducted in symptomatic patients undergoing gastroenterological examination at the Faculty of Medicine, University of Malaya, Kuala Lumpur, Malaysia. All biopsies were obtained with the informed consent of the patients and this study was approved by the Human Ethics Committees of the University of New South Wales and the University of Malaya. Based on endoscopic and histological examinations, patients were diagnosed as having gastric cancer or functional dyspepsia. All except seven samples were from patients with functional dyspepsia as shown in Table 2.

*H. pylori *was cultured by inoculating biopsies on *Campylobacter *selective agar (CSA) containing 4% blood base agar No. 2 (Oxoid), defibrinated horse blood (Oxoid), and one vial of Skirrow's supplement (Oxoid) containing 2.5 mg Trimethoprim, 5.0 mg Vancomycin, and 1250 IU polymyxin B. Primary cultures were incubated at 37°C with 10% CO_2 _in a CO_2 _incubator (Plymouth, USA) for up to 10 days, observing daily for growth. For isolation of pure cultures a single colony was picked and subcultured onto CSA for four days. Identification of *H. pylori *was based on microscopic morphology and biochemical testing (urease, oxidase and catalase). One isolate from each biopsy was selected for this study and 78 isolates were obtained from patients of different ethnic background, including 27 Chinese, 35 Indian and 16 Malay (Table 2). We used all Malay biopsy samples available. Despite the fact that this study spanned a period of four years the number of Malay subjects from whom *H. pylori *could be cultured was low which reflects the relative low prevalence in this population. Isolates from this study are available to researchers upon request to HM.

### Chromosomal DNA purification

One plateful of bacterial culture was collected and suspended into 215 μl of Tris (50 mM), 15 μl of EDTA (0.5 M) and incubated for 10 min. Two μl of proteinase K (10 mg/ml) and 20 μl of SDS (10%) were added followed by incubation at 50°C for a minimum of 2 h or until clear. One μl of RNase (10 mg/ml) was added and incubated at 65°C for an additional 20 min. the mixture was then transferred into a 1.5 ml Heavy Gel Phase tube (Eppendorf) and washed twice with one volume of phenol:chloroform:isoamyl alcohol (25:24:1) and once with one volume of chloroform:isoamyl alcohol (24:1). The top layer was transferred into a new 1.5 ml tube containing 600 μl of pre-chilled EtOH (100%). Precipitated DNA was then spooled out, washed in 70% (v/v) EtOH, dissolved in 100 μl TE buffer (10 mM Tris 1 mM EDTA, pH 8.0) and incubated at 65°C for 15 min to evaporate the residual ethanol.

### PCR assay and DNA sequencing

The primer sequences for MLST of the seven house keeping genes used in this study were those described by Achtman *et al*. [10]. Primers were synthesized commercially (Sigma-Aldrich). Each PCR reaction included 2.0 μl DNA template (approx. 20 ng), 0.5 μl (30 pmol/μl) of each forward and reverse primer, 0.5 μl of dNTP (10 mM), 5 μl of 10 × PCR buffer (500 mM KCl, 100 mM Tris-HCl, pH 9.0, 1% Triton X-100 and 15 mM MgCl_2_), 0.25 μl of *Taq *polymerase (1.25 U) and MilliQ water to a total volume of 50 μl. PCR cycles were performed in a Hybaid PCR Sprint Thermocycler (Hybaid): initial DNA denaturation for 2 min at 94°C, followed by DNA denaturation for 15 sec at 94°C, primer annealing for 30 sec at 50°C, and polymerization for 90 sec at 72°C for 35 cycles, with a final extension of 5 min at 72°C. PCR products were verified on ethidium bromide stained agarose gels. PCR product for sequencing was purified using sodium acetate/ethanol precipitation. The 20-μl PCR sequencing mixture contained 1 μl of BigDye (version 3.1; Applied Biosystems), 20 ng of the purified PCR product, 3.5 μl of 5× PCR sequencing buffer (Applied Biosystems), 1 μl of forward primer (concentration, 3.2 pmol/μl; Sigma-Aldrich), and MilliQ water. Unincorporated dye was removed by ethanol precipitation. The sequencing reaction mixtures were resolved on an ABI 3730 automated DNA sequence analyzer (Applied Biosystems) at the sequencing facility of the School of Biotechnology and Biomolecular Sciences, University of New South Wales, Sydney, Australia.

### Bioinformatic analysis

PHRED PHRAP and CONSED [37] program package, accessed through the Australia National Genomic Information Service, was used for sequence editing. PILEUP from the Genetics Computer Group package [38], and MULTICOMP [39], were used for multiple sequence alignment and comparison. PHYLIP [40] was used to generate phylogenetic trees. STRUCTURE version 2.2 [25], which implements a Bayesian approach for deducing population structure from multilocus data, was used to analyse the population clustering of an isolate, assuming that each isolate has derived all of its ancestry from only one population. The number of populations, *K*, was determined under the "no admixture" model and in each simulation run, the Markov Chain Monte Carlo (MCMC) simulation of 30,000 iterations approximated the posterior probability of *K*, following a burn-in of 10,000 iterations. After multiple runs on each *K *assumed, the value that generated the highest posterior probability was used as the number of possible populations. The assignment of an isolate to a particular population was done under the linkage model.

### Segregation site Analysis

Gene sequences from two populations were aligned and compiled by PILEUP and MULTICOMP. Informative sites, which are defined as those with at least two variants at a particular site and more than one isolate for each base variant, were extracted from output generated by MULTICOMP and examined using Microsoft EXCEL. Total base changes at each informative site present in each population were summed and formed a 2 × 2 table for Fisher's Exact test using SPSS (SPSS Inc, Chicago, IL). For those informative sites that have more than two variants, the least frequent base was removed and treated as a missing value. The probability of each site generated by SPSS was adjusted using Dunn-Sidak correction: *α*' = 1 - (1 - *α*)^1/*p*^, where α' represent adjusted probability, α represent the significance value (0.05 used in this study) and p represent the total number of comparisons.

The GenBank accession numbers for the sequences reported in this study are FJ846683 – FJ847228.

## Authors' contributions

RL conceived the study. CYT performed acquisition and analysis of data. HM and IWD participated in its design and coordination. QD participated in data acquisition. KLG contributed to the materials. All authors participated in drafting the manuscript, and read and approved the final manuscript.

## Supplementary Material

Additional file 1 **STRUCTURE analysis of Malaysian and global isolates**. The data provided represent the population structure of global isolates and the distribution of Malaysian isolates. Click here for file
