# Supplementary material for: Population structure of Helicobacter pylori among ethnic groups in Malaysia: recent acquisition of the bacterium by the Malay population
Source: BMC Microbiol. 2009 Jun 19;9:126. doi: 10.1186/1471-2180-9-126 (PMC2708179; doi:10.1186/1471-2180-9-126)
Supplement: Additional file 1 — STRUCTURE analysis of Malaysian and global isolates. The data provided represent the population structure of global isolates and the distribution of Malaysian isolates. [file 1471-2180-9-126-S1.pdf]

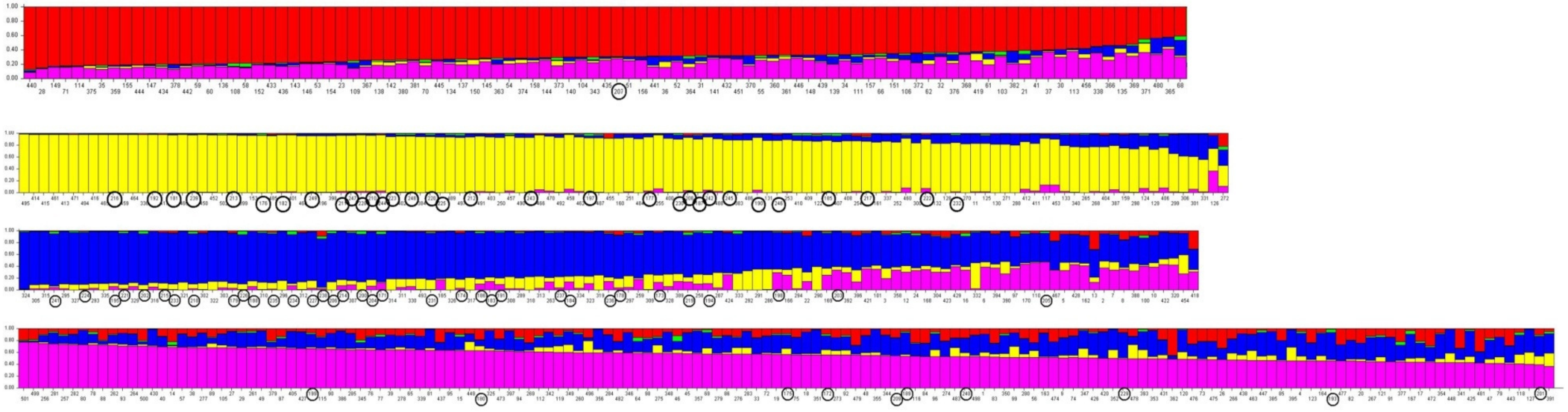

**STRUCTURE analysis of Malaysian and global isolates.** Population assignments were shown as 4 strips. Each strip is a population with colour codes for different populations (blue, purple, red and yellow for hpAsia2, hpEurope, hpAfrica1 and hpEastAsia respectively). Isolates from this study were circled under the population strip. Each vertical bar represents an isolate. Mosaic colours for an isolate indicate mixed population origin from respective populations of matching colour. Y-axis represents percentage of population assignment.
